# Supplementary material for: Loneliness Is Associated with Depressive Affect, But Not with Most Other Symptoms of Depression in Community-Dwelling Individuals: A Network Analysis
Source: Int J Environ Res Public Health. 2021 Mar 1;18(5):2408. doi: 10.3390/ijerph18052408 (PMC7967763; doi:10.3390/ijerph18052408)
Supplement: Supplementary file 1 [file ijerph-18-02408-s001.pdf]

Table S1. Associations with overall Patient Health Questionnaire-9 score with loneliness treated as a continuous variable.

Effect	Estimate	*SE*	95% CI		*p*
			LL	*UL*	
Intercept	3.69	0.388	2.93	4.45	<0.001
Gender (f)	0.81	0.217	0.38	1.23	<0.001
Age	−0.00	0.009	−0.02	0.02	0.792
Children	0.47	0.230	0.02	0.92	0.040
Sleep (h)	−0.02	0.017	−0.06	0.01	0.157
Loneliness	3.84	0.145	3.56	4.13	<0.001

Values are unstandardized beta coefficients with standard error (SE) and 95% confidence interval (CI); f, female; h, hours; F-statistic: 149.1 on 5 and 2001 DF, p-value: < 0.001; Adjusted R2 overall = 0.270; adjusted R2 change due to Loneliness = 0.261.

Table S2. Association of PHQ-2 (Feeling down, depressed, or hopeless) with loneliness.

Effect	Estimate	*SE*	95% CI		*p*
			LL	*UL*	
Intercept	−0.03	0.050	−0.13	0.07	0.558
Gender (f)	−0.01	0.027	−0.07	0.04	0.594
Age	−0.00	0.001	−0.00	0.00	0.444
Children	0.02	0.028	−0.03	0.08	0.415
Sleep (h)	−0.00	0.002	−0.00	0.00	0.739
PHQ_1	0.28	0.019	0.24	0.31	<0.001
PHQ_3	0.04	0.015	0.01	0.07	0.006
PHQ_4	0.10	0.018	0.07	0.14	<0.001
PHQ_5	0.04	0.017	0.00	0.07	0.031
PHQ_6	0.17	0.017	0.14	0.20	<0.001
PHQ_7	0.02	0.017	−0.01	0.05	0.240
PHQ_8	0.01	0.024	−0.04	0.06	0.696
PHQ_9	0.35	0.035	0.28	0.41	<0.001
L_some[a]	0.20	0.028	0.15	0.26	<0.001
L_often[b]	0.49	0.044	0.40	0.57	<0.001

Values are unstandardized beta coefficients with standard error (SE) and 95% confidence interval (CI); f, female; h, hours; loneliness treated as a continuous variable; [a]L_some = Dummy variable encoding loneliness "sometimes " as 1, "never" or "often" as 0; [b]L_often = Dummy variable encoding loneliness "often " as 1, "never" or "some-times" as 0; F-statistic: 191.5 on 12 and 1994 DF, p-value: < 0.001; Adjusted R2 overall = 0.53.

Table S3. Association of age and age^2 with loneliness.

Effect	Estimate	*SE*	95% CI		*p*
			LL	*UL*	
Intercept	1.17	0.164	0.85	1.49	<0.001
Gender (f)	0.06	0.033	0.00	0.13	0.062
Age	−0.03	0.009	−0.04	−0.01	0.005
Age^2	−0.00	0.000	0.00	0.00	0.043
Children	−0.08	0.037	−0.15	−0.01	0.025
Sleep (h)	−0.00	0.003	−0.01	0.00	0.107

Values are unstandardized beta coefficients with standard error (SE) and 95% confidence interval (CI); f, female; h, hours; loneliness treated as a continuous variable; F-statistic: 11.72 on 5 and 2001 DF, p-value: < 0.001; Adjusted R2 overall = 0.03.

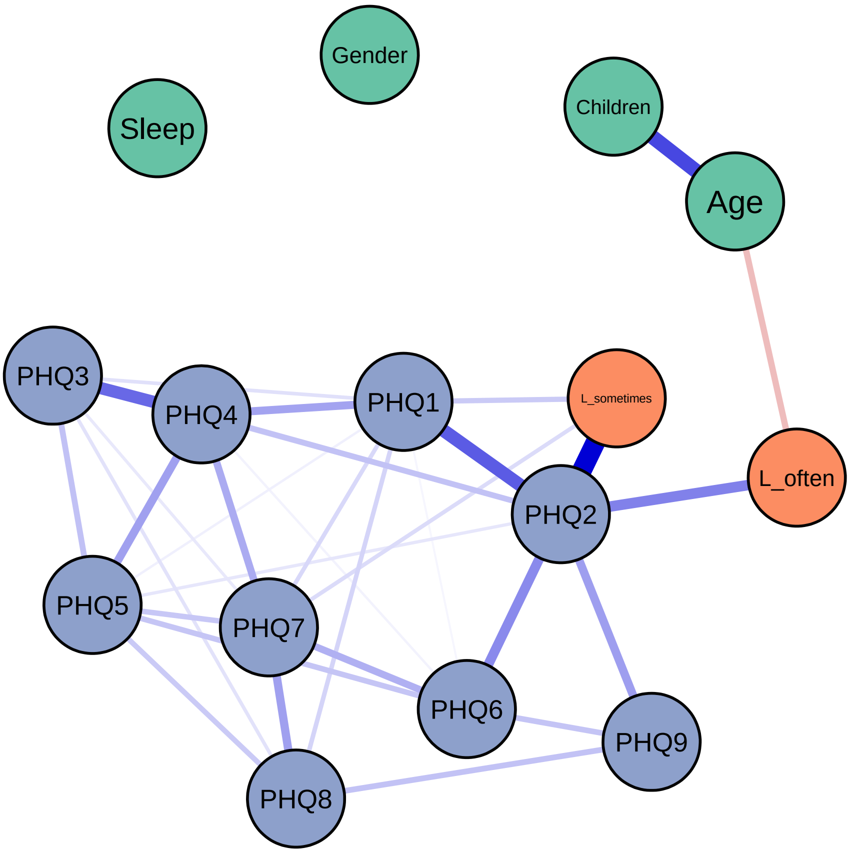

Figure 1. Visualization of Figure 1 using the Fruchterman-Reingold algorithm.

Relationships between PHQ-9 items, demographics and loneliness. Green: Demographics; Orange: Loneliness; Blue: PHQ; The edge between L_sometimes and L_often was excluded. L_sometimes = Dummy variable encoding loneliness “sometimes” as 1, “never” or “often” as 0; L_often = Dummy variable encoding loneliness “often” as 1, “never” or “sometimes” as 0; Children = living with children in the same household; Sleep = average hours of sleep per night in the previous 7 days; Patient Health Questionnaire (PHQ)-1 = Little interest or pleasure in doing things; PHQ-2 = Feeling down, depressed, or hopeless, ; PHQ-3 = Trouble falling or staying asleep, or sleeping too much; PHQ-4 = Feeling tired or having little energy; PHQ-5 = Poor appetite or overeating; PHQ-6 = Feeling bad about yourself – or that you are a failure or have let yourself or your family down; PHQ-7 = Trouble concentrating on things, such as reading the newspaper or watching television; PHQ-8 = Moving or speaking so slowly that other people could have noticed. Or the opposite – being so figety or restless that you have been moving around a lot more than usual; PHQ-9 = Thoughts that you would be better off dead, or of hurting yourself.

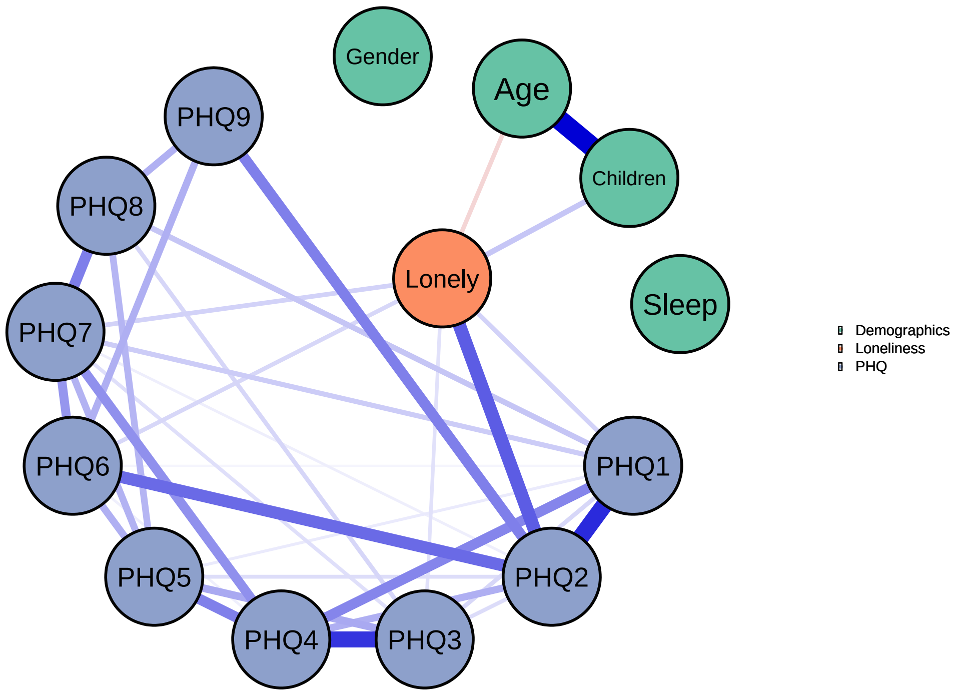

Figure S2. Relationships between Patient Health Questionnaire-9 items, demographics and loneliness treated as a continuous variable.

Children = living with children in the same household; Sleep = average hours of sleep per night in the previous 7 days; Patient Health Questionnaire (PHQ)-1 = Little interest or pleasure in doing things; PHQ-2 = Feeling down, depressed, or hopeless, ; PHQ-3 = Trouble falling or staying asleep, or sleeping too much; PHQ-4 = Feeling tired or having little energy; PHQ-5 = Poor appetite or overeating; PHQ-6 = Feeling bad about yourself – or that you are a failure or have let yourself or your family down; PHQ-7 = Trouble concentrating on things, such as reading the newspaper or watching television; PHQ-8 = Moving or speaking so slowly that other people could have noticed. Or the opposite – being so figety or restless that you have been moving around a lot more than usual; PHQ-9 = Thoughts that you would be better off dead, or of hurting yourself.

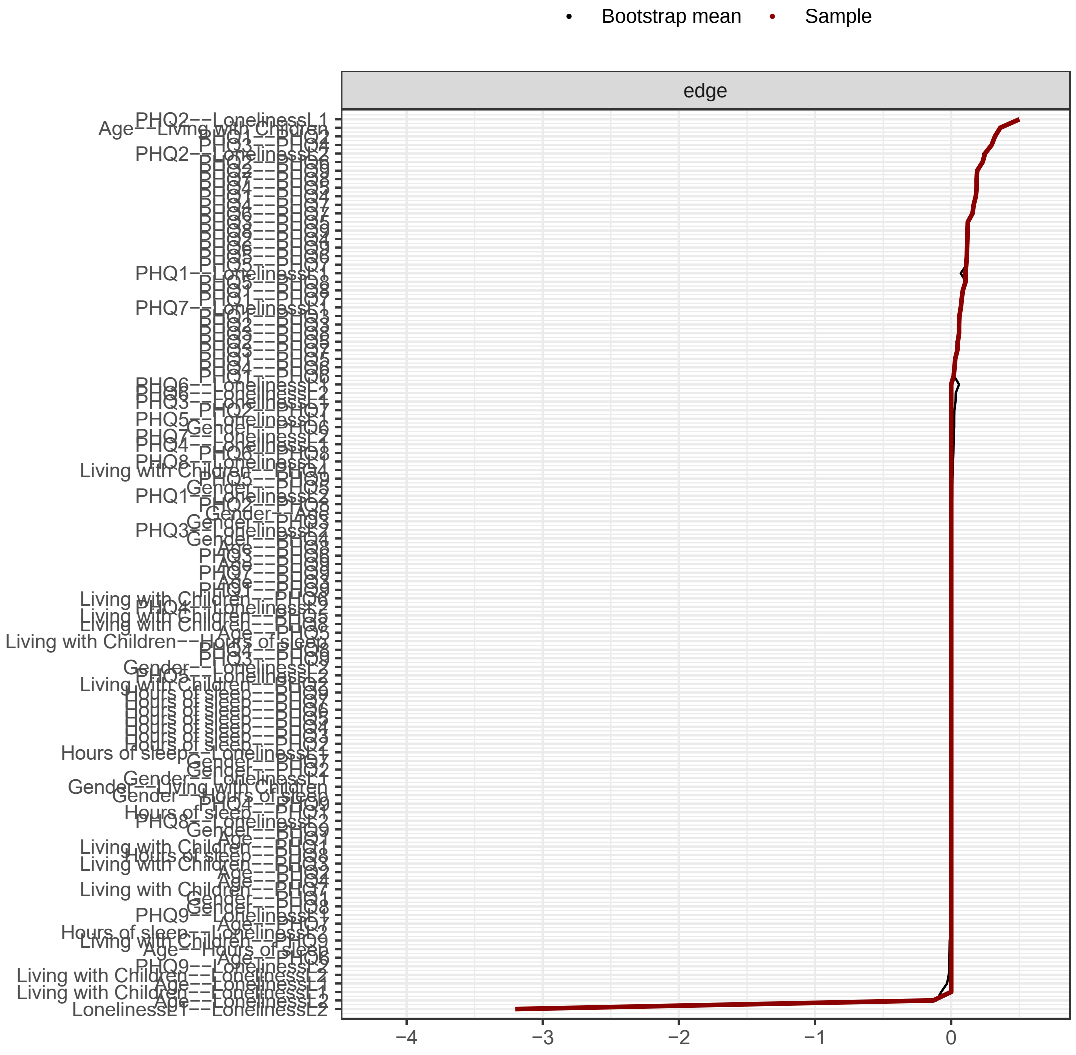

Figure S3. Bootstrap 95% confidence intervals of the edge weights of the network shown in Figure 1.

Loneliness represented by dummy variables. A horizontal line represents an individual edge. The red line indicates an individual edge's weight. The grey area represents the 95% confidence interval. L_sometimes = Dummy variable encoding loneliness "sometimes" as 1, "never" or "often" as 0; L_often = Dummy variable encoding loneliness "often" as 1, "never" or "sometimes" as 0; Children = living with children in the same household; Sleep = average hours of sleep per night in the previous 7 days; Patient Health Questionnaire (PHQ)-1 = Little interest or pleasure in doing things; PHQ-2 = Feeling down, depressed, or hopeless, ; PHQ-3 = Trouble falling or staying asleep, or sleeping too much; PHQ-4 = Feeling tired or having little energy; PHQ-5 = Poor appetite or overeating; PHQ-6 = Feeling bad about yourself – or that you are a failure or have let yourself or your family down; PHQ-7 = Trouble concentrating on things, such as reading the newspaper or watching television; PHQ-8 = Moving or speaking so slowly that other people could have noticed. Or the opposite – being so fidgety or restless that you have been moving around a lot more than usual; PHQ-9 = Thoughts that you would be better off dead, or of hurting yourself.

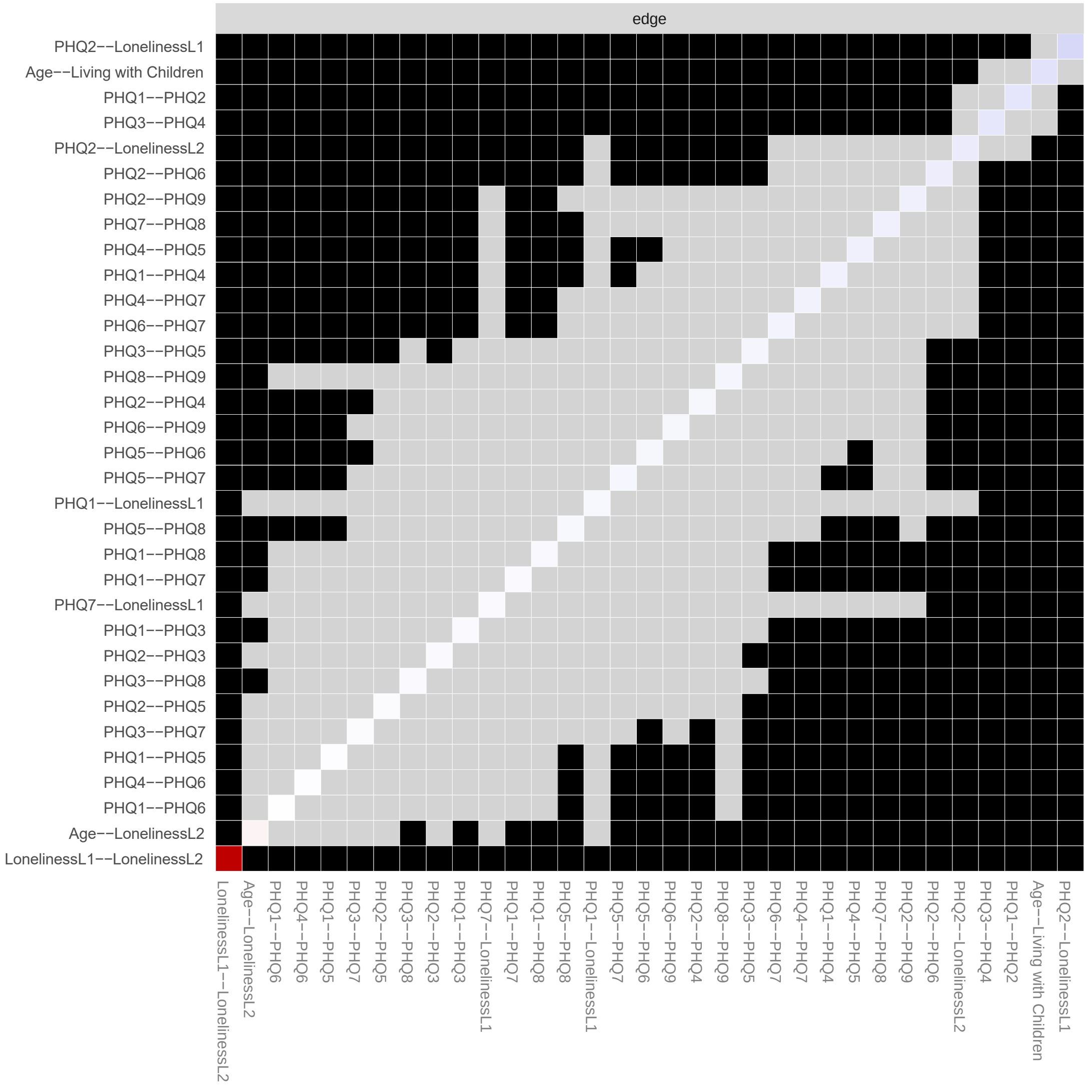

Figure S4. Bootstrap edge weights difference test between non-zero estimated edge-weights in the network shown in Figure 1.

Significant differences between non-zero estimated edges are indicated by black boxes. Non-significant differences are indicated by grey boxes. The colour of the diagonal boxes (ranging from red to blue) corresponds to the magnitude of the edge (negative to positive). L_sometimes = Dummy variable encoding loneliness "sometimes" as 1, "never" or "often" as 0; L_often = Dummy variable encoding loneliness "often" as 1, "never" or "sometimes" as 0; Children = living with children in the same household; Sleep = average hours of sleep per night in the previous 7 days; Patient Health Questionnaire (PHQ)-1 = Little interest or pleasure in doing things; PHQ-2 = Feeling down, depressed, or hopeless, ; PHQ-3 = Trouble falling or staying asleep, or sleeping too much; PHQ-4 = Feeling tired or having little energy; PHQ-5 = Poor appetite or overeating; PHQ-6 = Feeling bad about yourself – or that you are a failure or have let yourself or your family down; PHQ-7 = Trouble concentrating on things, such as reading the newspaper or watching television; PHQ-8 = Moving or speaking so slowly that other people could have noticed. Or the opposite – being so fidgety or restless that you have been moving around a lot more than usual; PHQ-9 = Thoughts that you would be better off dead, or of hurting yourself.
